# The Thermodynamic and Kinetic Properties of 2-Hydroxypyridine/2-Pyridone Tautomerization: A Theoretical and Computational Revisit

**DOI:** 10.3390/ijms17111893

**Published:** 2016-11-14

**Authors:** Safiyah A. Hejazi, Osman I. Osman, Abdulrahman O. Alyoubi, Saadullah G. Aziz, Rifaat H. Hilal

**Affiliations:** 1 Chemistry Department, Faculty of Science, King Abdulaziz University, P.O. Box 80203, Jeddah 21589, Saudi Arabia; shegazi@hotmail.com (S.A.H.); oabdelkarim@kau.edu.sa (O.I.O.); aalyoubi@kau.edu.sa (A.O.A.); saziz@kau.edu.sa (S.G.A.); 2 Chemistry Department, Faculty of Science, University of Khartoum, P.O. Box 321, Khartoum 11111, Sudan; 3 Chemistry Department, Faculty of Science, Cairo University, Cairo 11258, Egypt

**Keywords:** 2-hydroxypyridine, 2-pyridone, tautomerization, NBO, NLO

## Abstract

The gas-phase thermal tautomerization reaction between 2-hydroxypyridine (2-HPY) and 2-pyridone (2-PY) was investigated by applying 6-311++G** and aug-cc-pvdz basis sets incorporated into some density functional theory (DFT) and coupled cluster with singles and doubles (CCSD) methods. The geometrical structures, dipole moments, HOMO-LUMO energy gaps, total hyperpolarizability, kinetics and thermodynamics functions were monitored against the effects of the corrections imposed on these functionals. The small experimental energy difference between the two tautomers of 3.23 kJ/mol; was a real test of the accuracy of the applied levels of theory. M062X and CCSD methods predicted the preference of 2-HPY over 2-PY by 5–9 kJ/mol; while B3LYP functional favoured 2-PY by 1–3 kJ/mol. The CAM-B3LYP and ωB97XD functionals yielded mixed results depending on the basis set used. The source of preference of 2-HPY is the minimal steric hindrance and electrostatic repulsion that subdued the huge hyperconjugation in 2-PY. A 1,3-proton shift intramolecular gas-phase tautomerization yielded a high average activation of 137.152 kJ/mol; while the intermolecular mixed dimer interconversion gave an average barrier height of 30.844 kJ/mol. These findings are boosted by a natural bond orbital (NBO) technique. The low total hyperpolarizabilities of both tautomers mark out their poor nonlinear optical (NLO) behaviour. The enhancement of the total hyperpolarizability of 2-HPY over that of 2-PY is interpreted by the bond length alternation.

## 1. Introduction

2-Hydroxypyridine (2-HPY) is an aromatic heterocyclic compound. It represents an important class of compounds, which have π- and n-electrons systems. It tautomerizes to form 2-pyridone (2-PY) by a proton transfer between the nitrogen and oxygen atoms [1]. The tautomerism between 2-hydroxypyridine and 2-pyridone has been confirmed by X-ray [2], IR [3,4,5], UV [6], and microwave spectroscopy [7], together with some theoretical studies [8,9,10]. 2-Pyridones play important roles in bioactive systems and in current medicinal chemistry. They exhibit different pharmacological activities such as antipyretic, anti-inflammatory, analgesic, antimicrobial, b-adrenolytic, and antihypertensive activities. In addition, 2-pyridones are important intermediates in preparing different kinds of heterocyclic compounds, and they are flexible synthetic intermediates for the synthesis of alkaloids, drugs, and herbicides. 

The neutral 2-hydroxypyridine/2-pyridone tautomerism was widely studied [11,12]. The gas-phase internal energy difference between the two tautomers is ca. 3 kJ/mol in favor of the enol form [7]. In water, Aue et al. [13] reported an equilibrium constant of 900 and internal energy of 12 kJ/mol in favour of 2-PY. In cyclohexane, both tautomers coexist in comparable amounts [14]. 2-pyridone is predominantly favoured in the solid state [2,15,16] and polar solvents [17,18]. 

Different calculation methods were used to explore the tautomeric equilibrium of 2-hydroxypyridine/2-pyridone systems in the gas-phase [5]. AM1 [9] and MNDO [19] methods give results in quantitative agreement with the experimentally determined relative stability of gas-phase tautomers. MP2/6-31G** level of theory gave free energy changes for 2-PY in the gas-phase, cyclohexane and acetonitrile of −0.64, 0.33 and 9.28 kJ/mol in excellent agreement with experimental values [20]. Parchment et al. [21] obtained an energy difference of 2.9 kJ/mol between the two gas-phase tautomers using a large basis set (TZV2P) and electron correlation at the QCISD(T) level. Scanlan et al. [22] overestimated (10 kJ/mol) the relative stability of 2-hydroxypyridine (2-HPY) and 2-PY using ab initio calculations in a 3–21 G basis set. However, Hall et al. [23] using density functional theory (DFT) methods with gradient corrections predicted 2-PY being more stable than 2-HPY in disagreement with experimental [7] and some theoretical studies [9,19,20,22]. 

Numerous efforts devoted to studying the mechanism of the tautomeric equilibrium between 2-HPY and 2-PY. A semi-empirical (CNDO/2) calculation predicted a barrier height of 296 kJ/mol for the single molecular 1,3-proton tautomerization [24]. This activation energy approximates to 206 kJ/mol using a Hartree–Fock (HF)/3-21G level of theory [25]. Merino and Miller [26], applying configuration interaction with double zeta plus polarization functions, obtained a reduced barrier height of 160.67 kJ/mol. The existence of 2-HPY:2-PY dimeric forms was confirmed experimentally by infra-red (I.R.) spectroscopy [27]. Scanlan and Hillier [28] obtained a low barrier height of 46 kJ/mol for a self-associated mixed tautomer using SCF method with mixed basis sets. 

It is apparent from the literature tour that the predominance of the enol form over its keto counterpart is experimentally unambiguous. However, there have been many contradictory theoretical attempts; some in favour of the experimental findings, while others proved the contrary. In this paper, we try to revisit this issue quantum mechanically using some sophisticated methods with large basis sets to study the thermodynamic and kinetic stabilities of the 2-hydroxypyridine-2-pyridone system. We also shed light on the supposedly single molecular 1,3-proton transfer forbidden tautomerism. We employed the natural bond orbital technique to support our conjectures. 

## 2. Results and Discussion

### 2.1. Geometry

**Figure 1:**
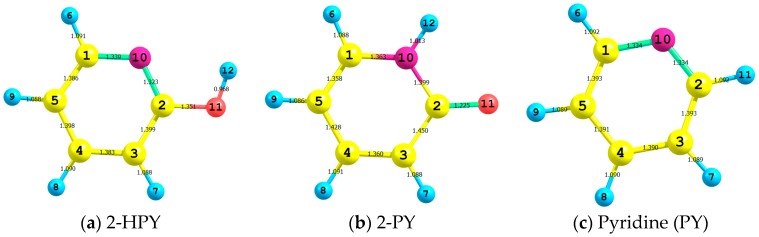
The atom numbering and bond lengths of (**a**) 2-hydroxypyridine (2-HPY); (**b**) 2-pyridone (2-PY); and (**c**) Pyridine which have been obtained at the CAM-B3LYP/aug-cc-pvdz level of theory. The color scheme is pink: nitrogen; yellow: carbon; blue: hydrogen; and red: oxygen.

In Figure 1 we show the atom numbering and the bond lengths of the optimized structures of gas-phase 2-hydroxypyridine (2-HPY), 2-pyridone (2-PY) and pyridine (PY) (see their standard coordinates shown in Supplementary Materials Tables S1–S3, respectively) which are obtained by using CAM-B3LYP [29] with aug-cc-pvdz [30] basis set. We preferred this level of theory because it gives the most satisfactory calculated geometrical values [31]. Some selected geometrical parameters of 2-HPY and 2-PY molecules obtained by B3LYP [32,33], CAM-B3LYP [29], ωB97XD [34], M062X [35] and coupled cluster with singles and doubles (CCSD) [36] methods with 6-311++G** [37,38,39] and aug-cc-pvdz [30] basis sets are listed in the Supplementary Materials (Table S4). In Table S4, the theoretical values for 2-PY are compared with the experimental crystal structure ones [2]. On the one hand, all the calculated O_11_C_2_ or N_10_C_2_ bond lengths are shorter or longer compared to their experimental rival by not more than 2.56% or 2.32%, respectively. On the other hand, the C_2_C_3_ and N_10_C_1_ bond lengths are quite close to their experimental counterparts with 1.04% and 1.25% deviations, respectively. The apparent deviation between the calculated and experimental structural parameters could be, in part, due to phase difference, i.e., the theoretical values are for gas-phase molecules, while the experimental ones are for a solid-state compound [2]. The ring N_10_C_2_C_3_ and C_2_C_3_C_4_ angles deviated by no more than 2° and 0.6°, respectively; while the outer ring O_11_C_2_C_3_ angles are wider by no more than 2.2°, compared to their peers. Both molecules are perfectly planar as indicated by 180° or 0.0° dihedral angles. This is supported by skeletal angles around 120° that result from sp^2^ hybridization. The shortening of the C_1_N_10_ and C_2_N_10_ bond lengths by 0.102 and 0.055 Å, respectively, compared to that of dimethyl amine [40], also complements these findings, indicating that these bonds have multiple bond characters. The optimized structures using the elected levels of theory for 2-HPY were compared with a normalized gas-phase microwave geometry [7] (See Table S4). Generally, excellent agreement between our calculated values and the microwave-normalized geometry was obtained. In particular, the CCSD method using both basis sets has reproduced the O_11_C_2_C_3_ angle and predicted the N_10_C_2_C_3_ and C_2_C_3_C_4_ angles with 0.3% and 0.2% errors, respectively. Apart from the predicted value of C_2_C_3_ bond, using 6-311++G** basis set, being shorter than its experimental [7] counterpart, all other bond lengths estimated by both basis sets are found to be longer than their experimental [7] peers, with errors ranging between 0.2% and 2%.

**Figure 2:**
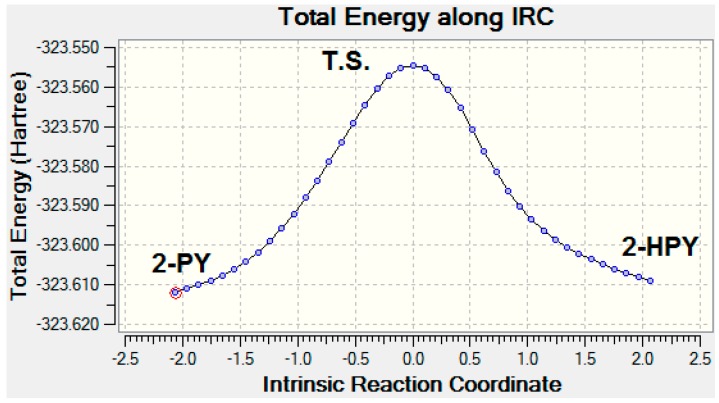
Intrinsic reaction coordinate (IRC) of the tautomerization of 2-HPYand 2-PY through the transition states (T.S.) which were obtained by using B3LYP/6-311++G** level of theory.

In the process of tautomerization of 2-HPY to form 2-PY, the C_2_O_11_ bond length shortened by 0.126 Å, while the C_2_C_3_ and N_10_C_2_ bond lengths elongated by 0.051 and 0.076 Å, respectively. In addition, the O_11_C_2_C_3_ angles opened up by ca. 8.1°; while the N_10_C_2_C_3_ angles closed up by ca. 10.6° (See Figure 1). The optimized structures of 2-HPY that tautomerized to 2-PY through the transition state (T.S.) (See Table S5 in the Supplementary Materials) which have been obtained by using CAM-B3LYP/aug-cc-pvdz level of theory are listed in Table 1 and pictured in Figure 2. The extracted remarks of a 1,3-proton shift process include: (1) The O_11_H_5_ bond length of 0.968 Å in 2-HPY elongated by 0.398 Å in the T.S. and eventually dissociated to release a proton. The proton then migrated and approached N_1_ by a distance of 1.290 Å before forming the N_10_H_5_ bond (1.013 Å) in 2-PY; (2) Consequently, the C_2_O_11_ bond abridged by 0.062 Å while C_2_N_10_ stretched by 0.032 Å in the T.S. and ultimately settled at 1.225 and 1.399 Å, respectively, in 2-PY; (3) The 2-HPY C_2_C_3_ bond length of 1.399 Å expanded by 0.008 Å in the T.S. and by a further 0.043 Å in 2-PY; (4) In concerted processes, the 2-HPY O_11_C_2_C_3_ and (N_10_C_2_C_3_) angles opened (closed) up by 15.8° (3.6°) in the T.S. and finally settling at 126.6° and (113.5°) in 2-PY; (5) The two tautomers together with the T.S. are perfectly planar; (6) No appreciable structural changes have been observed in remaining parts of the substrates.

### 2.2. Activation Energies

The zero-point electronic and activation energies of the tautomerization reaction: 2-HPY↔2-PY are listed in Table 2. These values were estimated by applying B3LYP, CAM-B3LYP, M062X and ωB97XD functionals with 6-311++G** and aug-cc-pvdz basis sets. Figure 2 illustrates the potential energy surface for this 1,3-proton transfer [41] tautomerization reaction exploiting B3LYP/6-311++G** level of theory. This level of theory has been picked because a noticeable harmony between the highly accurate Complete Basis Set (CBS) method and the B3LYP/6-311++G** level of theory was achieved for estimating the activation energies for triazoles and tetrazoles [42]. The analysis of the normal mode of the T.S. imaginary frequency (−1889.6 cm^−1^) revealed the journey of the migrating proton H5 between 2-HPY and 2-PY. (See Table S6 in the Supplementary Materials). The activation barriers for this gas-phase 1,3-proton transfer tautomerization reaction obtained by all elected levels of theory are comparable, with a mean value of ca. 137.152 kJ/mol. These barrier heights are in good agreement when compared with an activation energy of 153.6 kJ/mol calculated using CCSD and CCSD (T) methods [26]. It is worth noting that our relatively high gas-phase 1,3-proton transfer activation energies mean that the interconversion between 2-HPY and 2-PY could occur under high thermal conditions; or by proton tunneling [43]. However, we also studied the double proton shifts between a mixed dimer (See Figure S1 and Tables S7 and S8 in the Supplementary Materials) using the same levels of theory; where the activation energies were lowered by ca. four-folds to an average value of 30.84 kJ/mol (see Table 3). This is in excellent agreement with a value of 30.24 kJ/mol obtained by Tautermann et al. [43] using B3LYP/aug-cc-pvdz level of theory.

On the one hand, the activation energies, for the single-proton or the double-proton shift mechanisms, obtained by using 6-311++G** basis set were higher than the ones computed by the aug-cc-pvdz rival, regardless of the used DFT functional. On the other hand, the inclusion of the Coulomb-attenuating method with the exchange-correlation hybrid functional (CAM-B3LYP) [29] decreased the activation energies; while the use of the long-range corrected with empirical atom–atom dispersion corrections functional (ωB97XD) [34] increased the activation barriers in comparison with those obtained from the exchange-correlation hybrid functional (B3LYP) [32,33]. In addition, the hybrid Hartree–Fock exchange functional (M062X) [35] has yielded, comparatively, the lowest activation energies.

It would be instructive, at this point, to examine the effect of solvent polarity on the tautomerization reaction: 2-HPY↔2-PY. In water as a solvent, the more polar pyridone became a little bit more stable than the corresponding enol form by 18.103 kJ/mol. On the other hand, the transition state has been destabilized, thus increasing the activation barrier to 348.984 kJ/mol.

### 2.3. Therodynamic Analysis

Table 4 lists the values of ∆E°, ∆H°, ∆S°, ∆G° and K for the tautomerization reaction: 2-HPY↔2-PY computed by utilizing B3LYP, CAM-B3LYP, ωB97XD, M062X and CCSD methods with 6-311++G** and aug-cc-pvdz basis sets at 298.15 K. Regardless of the basis set used, the M062X and CCSD methods favour 2-HPY over 2-PY by 5–9 kJ/mol. The CCSD method calculated amounts are in good agreement with the microwave experimental value of 3.23 kJ/mole [7]; while the ones obtained from M062X overestimated it. Opposing to these results, 2-PY is favoured when applying B3LYP method with both basis sets; as shown by Hall et al. [23]. The embodiment of the long-range correction on the traditional hybrid B3LYP produced the CAM-B3LYP functional [29]. The influence of the long-range correction parameter on ∆E is seen when CAM-B3LYP is applied with aug-cc-pvdz and 6-311++G** basis sets. In these two cases, the inclusion of the long-range correction with aug-cc-pvdz basis set boosted the stability of 2-HPY over that of 2-PY by ca. 2 kJ/mol; while when 6-311++G** basis set is utilized the 2-PY tautomer is slightly favoured by less than one kJ/mol. The overall ambiguities observed in these calculations are due to the small total energy difference between the two tautomers, which is below the chemical accuracy limit of 4.184 kJ/mol, set for the theoretical calculations of total energies of chemical entities. That is, the energy difference is very sensitive to the accuracy of the method. Hence, any small error in total energy could lead to large deviation in energy difference or relative stability [44]. 

As shown in Table 4, the effect of the long-range correction on the exchange-correlation hybrid functional can be visualized by comparing ∆G values obtained from B3LYP and CAM-B3LYP methods with 6-311++G** and aug-cc-pvdz basis sets. It decreased ∆G by 2.523 kJ/mol when using 6-311++G** basis set and by 2.631 kJ/mol from aug-cc-pvdz; which implies respective increases of K by 0.404 and 1.274. These changes in the values of ∆G and K meant that the reaction: 2-PY↔2-HPY is moving toward being spontaneous with gradual increase of the relative concentration of 2-HPY. In addition, the modulation of damped atom–atom dispersion corrections on long-range corrected hybrid density functionals produces ωB97XD functional [34]. The impact of dispersion correction on ∆E, ∆G and K values are also shown in Table 4; where the results from CAM-B3LYP and ωB97XD functionals with 6-311++G** and aug-cc-pvdz basis sets are contrasted. It is clear that the dispersion correction elevated ∆E by 1.927 kJ/mol from 6-311++G** and by 1.728 kJ/mol from aug-cc-pvdz basis set. Consequently, the dispersion-correction appears in elevating ∆G when using both 6-311++G** and aug-cc-pvdz basis sets by 1.935 and 1.738 kJ/mol respectively; implying a decrease of equilibrium constants K at 298.15 K by 0.342 and 0.981, respectively. That is, the dispersion correction contradicted experimental findings [7]. In contrast, the thermodynamic functions predicted with hybrid Hartree–Fock exchange functional (M062X) [35], regardless of the basis set used, consolidated qualitatively fairly well the experimental observations [7]. As expected, the application of the electron correlation coupled cluster method has reproduced the earlier experimental [7] and theoretical studies [45]. Therefore, these results indicated that the accuracy of these methods in predicting the relative stabilities of these tautomers are in the order: CCSD > M062X > CAMB3LYP > ωB97XD > B3LYP.

All elected levels of theory gave ∆G values that are influenced mostly by ∆H (52%–85%) with moderately less contribution from T∆S (15%–48%). These percentage divergences are assigned to the characteristics of functionals as well as the effects of the basis sets used. The equilibrium constants values at 298.15 K varied greatly with the different levels of theory [5]. This is because the elected levels of theory predicted different stabilities for the two tautomers. The equilibrium concentration obtained by using CCSD/6-311++G** level of theory reproduced most closely the experimental value [7]. This meant that the equilibrium concentration of 2-HPY is ca. three to four times that of 2-PY. The highest equilibrium concentration for 2-HPY of more than 6-fold compared to that of 2-PY was obtained by using CCSD/aug-cc-pvdz level of theory. The M062X functional with the two basis sets greatly overestimated the equilibrium concentrations of 2-HPY over that of 2-PY. Finally, the negative values of ∆S showed that the preference of 2-HPY over 2-PY occurred at all temperatures.

### 2.4. Natural Bond Orbital (NBO) Analysis

**Figure 3:**
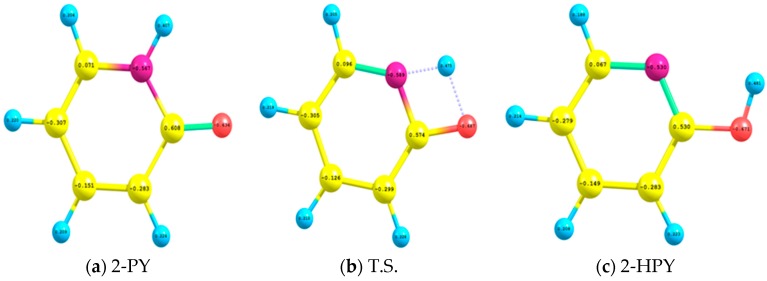
The natural atomic charges of (**a**) 2-hydroxypyridine (2-PY); (**b**) the transition state (T.S.); and (**c**) 2-pyridone (2-HPY) which were calculated by utilizing CAM-B3LYP/aug-cc-pvdz level of theory. For color scheme see caption of Figure 1.

In Figure 3 we depict the natural atomic charges of 2-PY, T.S. and 2-HPY which have been computed by applying B3LYP/aug-cc-pvdz level of theory. For 2-PY, the nitrogen and oxygen atoms N10 and O11 acquired negative charges of −0.567e and −0.636e, respectively; while the carbon atom C2 and the future dislodging hydrogen atom H12 developed positive charges of 0.608e and 0.407e, respectively. In the T.S., the natural atomic charges of the nitrogen and oxygen atoms (−0.589e and −0.687e) and the transiting proton (0.475e) increased a little bit; whilst that of C2 slightly decreased (0.574e). When H12 binds to O11 in the 2-HPY, the negative natural atomic charge of N10 decreased by 0.037e while that of O11 increased by 0.035e, compared to those of the 2-PY tautomer. Consequently, the positive natural charge of C2 diminished by 0.037e while that for H12 is maximized (0.481e). These natural charges are in good agreement with those reported by Hatherley et al. [7].

In Table 5 we list the second-order perturbation (E_(2)_) calculation of the hyperconjugative and hydrogen bonding energies of 2-HPY, T.S. and 2-PY which were estimated by applying CAM-B3LYP/aug-cc-pvdz level of theory. Natural Bond Orbital (NBO) theory [46,47] boosts the analysis of hydrogen bonding [48] and hyperconjugative [49] interactions through using the second-order perturbation (E_(2)_) technique given by:

(1) $$E_{\left(2\right)}=\Delta E_{ij}=\;\frac{q_{i}{\left(F_{ij}\right)}^{2}}{\Delta \varepsilon }$$

where $q_{i}$ refers to the occupancy of the donor orbital, $F_{ij}$ assesses the off-diagonal NBO Kohn–Sham Matrix elements and ∆ε evaluates the donor (i) and acceptor (j) orbital energy difference. With reference to Table 5, the most influential hyperconjugative π→π*, σ→σ* and n→σ*(π*) interactions for 2-HPY, T.S. and 2-PY are shown. On the one hand, the π→π* interactions contributed 694.0, 110.56 and 327.52 kJ/mol for the stabilization of 2-HPY, T.S. and 2-PY, respectively. The most strong of which is the π_C3–C4_→π*_C5–N10_ interaction in 2-HPY (163.76 KJ/mol) and 2-PY (128.72 kJ/mol); while the T.S. is strongly stabilized by π_C1–C5_→π*_C3–C4_ that availed 18.04 kJ/mol. On the other hand, the nitrogen and oxygen atoms lone pairs contributed totals of 81.93, 906.52 and 732.6 kJ/mol for the stabilization of 2-HPY, T.S. and 2-PY, respectively. The strongest of which are the n_2O11_→π*_C2-N10_ that availed 172.32 kJ/mol for the stability of 2-HPY and the n_1N10_→π*_C2-O11_ interaction that benefited the persistence of 2-PY by 66.97 kJ/mol. The stability of the T.S. is overwhelmingly enhanced by 401.44 kJ/mol from the n_2N10_→n*_H12_ interaction. It is worth noting that the 2-HPY tautomer is mostly stabilized by the π→π* interactions while the stability of 2-PY is mostly enhanced by the n→σ* or n→π* interactions; called the lone-par effect [50]. In addition, the very high charge transfer interactions n_2O11_→n*_1H12_ and n_1N10_→n*_1H12_ that yielded 284.44 and 401.44 kJ/mol for the stability of T.S., respectively, drove the debilitation of the O_11_H_12_ and N_10_H_12_ bonds and hence expediting the transport of the proton from O_11_ to N_11_ and vice-versa; through a 1,3-proton shift [41] mechanism.

Our elected levels of theory yielded contradictory relative stability of the two tautomers. This is because, experimentally, the relative stability between 2-HPY and 2-PY is in favour of the former by ca. 3.23 kJ/mol [7], which is less than the chemical accuracy limit of 4.184 kJ/mol [44]. To solve this problem, we embarked on applying a more rigorous approach that enforces the steric, electrostatic or hyperconjugative interactions [51] for the assertiveness of the relative stability. The relative influences of these three factors on the relative stability of 2-HPY and 2-PY were disbanded by using the natural bond orbital (NBO) technique that applies the $DEL Keylist of the NBO version 3.1 [52] which is integrated into the Gaussian09 Suite [53].

The results encompassing the total SCF, deletion and delocalization energies of 2-HPY and 2-PY using CAM-B3LYP/aug-cc-pvdz//CCSD/aug-cc-pvdz level of theory are listed in Table 6. The total SCF energy is symptomatic to the concerted action of the three factors; where 2-HPY is supported over 2-PY by 1.090 kJ/mol. The energy of deletion prognosticates the Lewis Structures of 2-HPY and 2-PY, in which only the steric and electrostatic factors dominate. On the one hand, the Lewis Structures portended the preference of 2-HPY over 2-PY by 80.582 kJ/mol because of minimal steric hindrance and smaller electrostatic repulsion in 2-HPY compared to their impact in the 2-PY tautomer. This is demonstrated by the congestion imposed by the shortening of the C2O11 bond by 0.126 Å and the presence of five hydrogen atoms attached to the pyridine ring compared to four of them in 2-HPY, which consequently increased the electrostatic repulsion in 2-PY relative to that in 2-HPY. On the other hand, the hyperconjugative interactions were competitive enough to release 79.492 kJ/mol for stabilization of 2-PY over 2-HPY. This is achieved mainly by the n_1N10_→π*_C1–C5_ and n_1N10_→π*_C2–O11_ interactions which profited 2-PY by 223.2 and 334.84 kJ/mol compared to 39.96 and 2.0 kJ/mol for 2-HPY. The energy enhancements of these interactions are brought about by the shortening of the C1C5 and C2O10 bonds in 2-PY by 0.028 and 0.126 Å, respectively, compared to their values in 2-HPY. This environment maximized the overlap between the lone pairs of the N10 and O11 atoms and the π*_C1–C5_ and π*_C2–O11_ bonds for the former tautomer compared to the latter one. Despite the prevailing hyperconjugative competitiveness of 2-PY compared to 2-HPY, it still could not surmount the overall stabilization of the latter imposed mainly by the steric and electrostatic factors. Finally, we can securely conclude that the preference of 2-HPY over 2-PY is mainly due to the comparatively minimum steric hindrance and electrostatic repulsion.

### 2.5. Nonlinear Optical (NLO) Properties

It is known that heterocyclic compounds have enhanced charge transfer, which might lead to large nonlinear optical properties. In the present section, we intend to test this information and avail any basic information related to the hyperpolarizability of these tautomers to the scientific community. Furthermore, we will present results of calculations, of nonlinear optical properties, at different levels of DFT theory and provide detailed discussion of the advantages and disadvantage of each functional used. The nonlinear optical (NLO) properties of 2-HPY and 2-PY which were calculated by using B3LYP, CAM-B3LYP, M062X and ωB97XD functionals with 6-311++G** and aug-cc-pvdz basis sets are listed in Table 7. The hyperpolarizabilities are given in atomic units (a.u.) which are related to electrostatic units (esu) through the conversion factor: 1 a.u. = 8.6393 × 10^−33^ esu. The total hyperpolarizabilities (β_tot_) are estimated by:

β_tot_ = (β_*x*_^2^ + β_*y*_^2^ + β_*z*_^2^)^½^

where β_*i*_ = β_*iii*_ + ⅓∑(β_*ijj*_ + β_*jij*_ + β_*jji*_)

The dipole moments (µ), the frontier orbitals (FMOs) (Highest Occupied Molecular Orbitals (HOMOs) and Lowest Unoccupied Molecular Orbitals (LUMOs)) energies together with the energy gaps (E.G.) of 2-HPY and 2-PY, which were computed by applying the aforementioned elected levels of theory, are listed in Table 7. For comparison purposes, some calculated [54] and measured [55] total hyperpolarizabilities for p-nitro-aniline (pNA), as a prototypical NLO compound, are also recorded. All elected levels of theory estimated the dipole moments of 2-PY to be ca. three times those of 2-HPY. Regardless of the method used, the 6-311++G** basis set computed larger dipole moments (4%–7%) compared to those obtained by aug-cc-pvdz basis set; with the ones estimated by the latter basis set approached closely (2%–4%) the experimental values [7]. It is apparent from Table 7 that the values of the dipole moments depend on the method used rather than the accompanying basis set.

The HOMO-LUMO energy gaps (E.G.) for 2-HPY were estimated to be higher than those for 2-PY by all elected levels of theory (Table 7). It can be seen that the traditional hybrid B3LYP method yielded lower values compared to those computed by the LC-DFT functionals (CAM-B3LYP and ωB97XD) and the global hybrid functional with 54% HF exchange (M062X). This means that the effect of the long-range correction [29], has increased the E.G. by 46%–68%; whilst the inclusion of dispersion correction [34] inflicted a further increase by 9%–19%. The implication of 54% HF on the DFT functional [35] has again brought about an E.G. rise of 41%–51%. It is noteworthy that the basis set effect is minimal (less than 1%) compared to that of the functionals; with the 6-311++G** basis set computing slightly higher values compared to those obtained from aug-cc-pvdz. 

**Figure 4:**
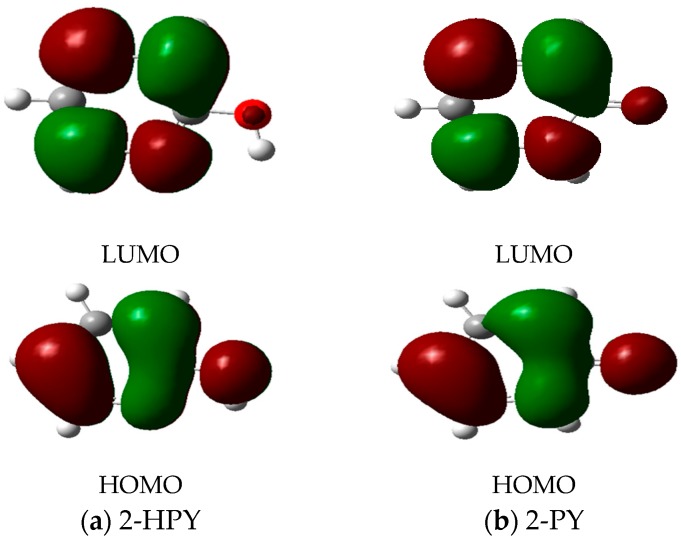
Frontier Molecular Orbitals of (**a**) 2-Hydroxyprydine (2-HPY) and (**b**) 2-pyridone (2-PY) which were calculated by using CAM-B3LYP/aug-cc-pvdz level of theory.

The frontier molecular orbitals (FMOs) of 2-HPY and 2-PY computed by applying B3LYP/6-311++G** level of theory are depicted in Figure 4. Their LUMOs are distributed mainly over the pyridine ring as π(C=N) and π(C=C) antibonding orbitals; while their HOMOs are delocalized also over the pyridine ring but as π(C=N) and π(C=C) bonding orbitals together with oxygen atom lone pairs. Before we take the discussion of MOs any further, let us emphasize that the Kohn–Sham (KS) MO’s resembles those obtained from a typical HF calculations. Cramer has provided a detailed discussion of this point [56]. Furthermore, an exposition of the concept of electronegativity as the negative average of the KS HOMO and LUMO energies and the chemical potential (χ) as lying midway the HOMO-LUMO energy gap has been discussed by Feller and Peterson [57].

**Figure 5:**
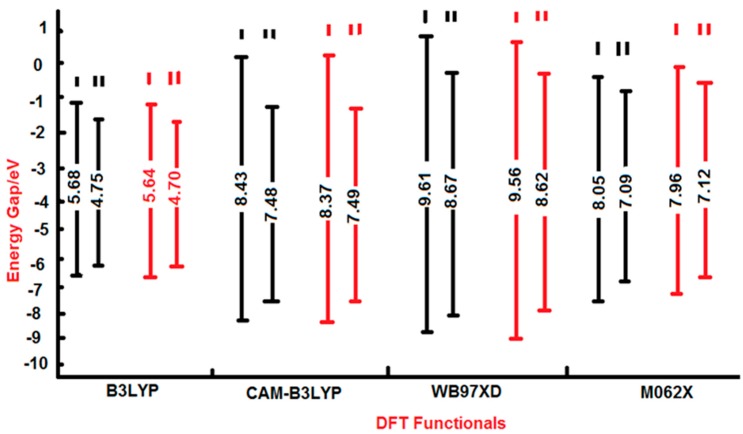
Schematic molecular orbital energy level diagram for the energy gaps of 2-HPY (I); and 2-PY (II) tautomers. It has been drawn using data from B3LYP, CAM-B3LYP, M062X and wB97XD functionals with 6-311++G** (black lines); and aug-cc-pvdz (red lines) basis sets.

A glimpse at Table 7 and Figure 5 shows that the traditional hybrid functional (B3LYP) destabilized the HOMOs but stabilized the LUMOs; rendering lower E.Gs. The application of CAM-B3LYP (long-range correction) and ωB97XD (dispersion correction) functionals has immensely stabilized the HOMOs and destabilized the LUMOs; leading to too high E.Gs. In contrast, the employment of M062X functional (54% HF) has little effect on FMOs compared to that of the B3LYP functional; yielding comparatively slightly higher E.Gs. The lucid impact on the energy gaps follows the sequence: ωB97XD > CAM-B3LYP > M062X > B3LYP. The aug-cc-pvdz basis set estimated slightly lower energy gaps compared to those computed by 6-311++G**. All these results are in adorable congruence with prior findings [58,59,60].

Table 7 lists the total hyperpolarizabilities (β_tot_) of 2-HPY and 2-PY which are computed by utilizing B3LYP, CAM-B3LYP, M062X and ωB97XD functionals with 6-311++G** and aug-cc-pvdz basis sets. Apart from the value obtained for 2-PY using M062X/aug-cc-pvdz level of theory and regardless of the basis set used, the β_tot_ values computed by the traditional hybrid functional (B3LYP) are generally higher than those obtained from M062X, CAM-B3LYP and ωB97XD functionals [59]. In contrast, the CAM-B3LYP functional yielded comparatively the lowest values, with the exception of that for 2-HPY obtained from M062XA/6-311++G** level of theory. It is interesting to note that the total hyperpolarizabilities of 2-HPY are higher than those of 2-PY by 4%–33%.

However, in comparison to the calculated [54] and measured [55] hyperpolarizabilities of pNA, our estimated β_tot_ values for 2-HPY and 2-PY are extremely small. Therefore, both the perfectly planar 2-HPY and 2-PY could not show any appreciable NLO properties. The reason could be due to the absence of an electron-donating group for a typical push-pull π-conjugated system [58]. Therefore, their NLO properties could supposedly be sutured and extremely consolidated by attaching any electron-donating group, opposite to the electron-withdrawing hydroxy or oxygen moieties.

It has been established theoretically [61] and in two independent studies by de Silva et al. [62,63] on novel charge transfer molecular systems and experimentally [64] that the total hyperpolarizabilities are inversely proportional to the energy gaps. That is, the smaller energy gaps encourage the eventuality of charge transfer, which in turn render higher NLO characters. As seen from Table 7, our elected tautomers infringe this criterion. It is noteworthy that a few other agents transcribe the boosted NLO characters. They comprise, in addition to small energy gaps, planarity, large dipole moments, bond length alternation, presence of electron withdrawing and donating groups and formation of H-bonding [65,66]. 2-HPY and 2-PY tautomers are devoid of a donor-acceptor push-pull system; which is the most influential factor in dictating the enhanced total hyperpolarizability. It is interesting to perceive that although 2-PY has smaller energy gaps and larger dipole moments compared to 2-HPY; the latter showed relatively a slightly higher total hyper polarizability. This is could be due to bond length alternation of the two tautomers compared with their parent pyridine. That is, the optimized geometries of 2-HPY, 2-PY and pyridine obtained by using CAM-B3LYP/aug-cc-pvdz level of theory (see Figure 1) showed that the C–C double bonds of 2-HPY get a bit longer while the single bonds become a bit shorter compared to those of 2-PY; leading to the enhancement of hyperpolarizability.

## 3. Computational Details

The ab initio quantum mechanical molecular orbital calculations were performed using the Gaussian09 program [53]. The structure and properties of the studied molecules were visualized by using GaussView [67] and Chemcraft [68] softwares. The transition state (T.S.) between 2-HPY and 2-PY was located with the Berry Keyword [69]. Intrinsic Reaction Coordinate (IRC) [70] monitored the potential energy profile for this tautomerization. A number of density functional theory (DFT) [71] and coupled cluster with singles and doubles (CCSD) [36] methods were tested. They are combined with the triple zeta and polarization functions at the hydrogen and carbon atoms (6-311++G**) [37,38,39] and augmented correlation consistent polarization with double zeta functions (aug-cc-pvdz) [30] basis sets to optimize the geometrical structure of these substrates together with their kinetic, thermodynamic and nonlinear optical (NLO) properties. The DFT functionals include the Becke, three-parameter, Lee-Yang-Parr exchange-correlation hybrid functional (B3LYP) [32,33], the Coulomb-attenuating method that combines the hybrid qualities of B3LYP and the long-range correction (CAM-B3LYP) [29], long-range corrected (LC) hybrid density functional with empirical atom–atom dispersion corrections (ωB97XD) [34] and the Minnesota hybrid functional with 54% Hartree–Fock (HF) exchange (M062X) [35]. A natural bond orbital (NBO) analysis was performed by using the NBO Program as implemented in Gaussian 09 software package. The influence of bulk solvent was accounted for by the CPCM polarizable conductor calculation model as implemented in Gaussian 09. In the CPCM model [72], the solvent is represented by a constant dielectric medium surrounding a cavity built around the solute.

## 4. Conclusions

DFT functionals and CCSD method with 6-311++G** and aug-cc-pvdz basis sets were utilized to investigate the thermal tautomerization of 2-hydroxypyrine (2-HPY) and 2-Pyridone (2-PY). The optimized structures of the title molecules are in excellent agreement with the experimental and theoretical findings. The geometrical changes trace correctly the route of conversion from 2-HPY to 2-PY through the transition state (T.S.). We obtained a relatively high average activation barrier of 137.2 kJ/mol for a unimolecular 1,3-proton transfer tautomerism. It is reduced to 30.84 kJ/mol for a mixed dimer tautomerization using the same levels of theory. Therefore, the single molecular tautomerization is forbidden at low temperature; but could be feasible under high temperature or through proton tunneling. The CCSD method favoured 2-HPY over 2-PY by a relative energy stability very close to the microwave experimental value; while M062X overestimated it and B3LYP functional gave a reversed stability. Thermodynamically, 2-HPY is favoured at all temperatures. The 2-HPY tautomer is mostly stabilized by π→π* interactions (173.50 kcal/mol); while 2-PY and T.S. are enhanced by n→π* and n→n* interactions that supported them by 183.15 and 100.36 kcal/mol, respectively. The comparatively minimum steric hindrance and electrostatic repulsion in 2-HPY vanquished the overwhelming hyperconjugative effect in 2-PY; leading to an overall relative stability of the former by 1.368 kJ/mol. The total hyperpolarizability of 2-HPY exceeds that of 2-PY despite the large dipole moment and smaller energy gap of the latter. The bond length alternation perceived to be the cause of the enhancement. 

Future analysis of the extent and type of delocalization in the studied pyridine derivatives can in principle be carried out using newly developed indices [73,74]. The first is based on the ability to derive colored representations of widely employed distance-based topological indices from chemical reactivity electronegativity and chemical hardness [73]. This has been developed in the novel theoretical tool, meaningful topo-reactive or structure-reactivity indices with application to polycyclic aromatic hydrocarbons (PAHs). The second index [74] presents a new definition of the softness kernel based on the exchange–correlation density. This new kernel is shown to correspond to the change of electron fluctuation upon external perturbation, thus helping to bridge the gap between conceptual density functional theory and some tools describing electron localization in molecules. 

## Figures and Tables

**Table 1:** Some selected optimized bond lengths of 2-hydroxypyridine (2-HPY) that tautomerized forming 2-Pyridone (2-PY) through the transition state (T.S.) which were obtained by using CAM-B3LYP/aug-cc-pvdz level of theory.

Parameter	2-HPY	T.S.	2-PY
C_2_O_11_	1.351	1.289	1.225
C_2_N_10_	1.323	1.355	1.399
C_2_C_3_	1.399	1.407	1.450
C_1_N_10_	1.339	1.336	1.363
O_11_H_5_	0.968	1.366	-
N_11_H_5_	-	1.290	1.013
O_11_C_2_C_3_	118.5	134.3	126.6
N_10_C_2_C_3_	124.1	120.5	113.5
C_2_C_3_C_4_	117.4	116.4	121.4

**Table 2:** The total zero-point energy (a.u.) of 2-HPY, T.S. and 2-PY forms and activation energies (kJ/mol) of 2-HPY and 2-PY tautomers using different functionals with 6-311++G** and aug-cc-pvdz basis sets.

Functional	Basis Set	2-HPY	T.S.	2-PY
B3LYP	6-311++G**	−323.5205	−323.4665	−323.5218
	Activ. Energy	135.544		138.080
	aug-cc-pvdz	323.4725	−323.4195	−323.4727
	Activ. Energy	133.032		133.536
CAM-B3LYP	6-311++G**	−323.3592	−323.3034	−323.3595
	Activ. Energy	140.06		140.812
	aug-cc-pvdz	−323.3102	−323.2555	−323.3094
	Activ. Energy	137.3		135.292
ωB97XD	6-311++G**	−323.4021	−323.3469	−323.4031
	Activ. Energy	138.556		141.064
	aug-cc-pvdz	−323.3591	−323.3049	−323.3590
	Activ. Energy	136.044		135.792
M062X	6-311++G**	−323.3834	−323.3258	−323.3810
	Activ. Energy	144.58		138.556
	aug-cc-pvdz	−323.3480	−323.2916	−323.3446
	Activ. Energy	141.568		133.032

**Table 3:** The total energy (a.u.) of the mixed dimer (2-HPY–2-PY) and the transition state (T.S.) and the activation energies (kJ/mol) for the double proton transfer of the 2-HPY and 2-PY tautomers using different DFT) functionals with 6-311++G** and aug-cc-pvdz basis sets.

Functional	Basis Set	2-HPY–2-PY	T.S.	Activ. Energy
B3LYP	6-311++G**	−647.2530	−647.2398	33.532
	aug-cc-pvdz	−647.1573	−647.1452	30.372
CAM-B3LYP	6-311++G**	−646.9344	−646.9214	32.632
	aug-cc-pvdz	−646.8367	−646.8249	29.62
ωB97XD	6-311++G**	−647.0232	−647.0091	35.392
	aug-cc-pvdz	−646.9371	−646.9239	33.532
M062X	6-311++G**	−646.9798	−646.9688	27.612
	aug-cc-pvdz	−646.9088	−646.8989	24.848

**Table 4:** The zero-point total energy (ΔE/kJ·mol^−1^), enthalpy (ΔH/kJ·mol^−1^), free energy (ΔG/kJ·mol^−1^) and entropy (ΔS/J·mol^−1^·K^−1^) changes and equilibrium constant (K) for the tautomerization reaction: 2-PY↔2-HPY at 298.15 K.

Level of Theory	ΔE	ΔH°	ΔG°	ΔS°	K
B3LYP/6-311++G**	52.72	50.32	58.56	−27.632	3.648
B3LYP/aug-cc-pvdz	10.416	8.576	15.632	−23.664	10.784
CAM-B3LYP/6-311++G**	12.096	9.536	18.192	−29.04	10.112
CAM-B3LYP/aug-cc-pvdz	−31.808	−33.648	−26.464	−24.096	31.168
M062X/6-311++G**	−102.496	−105.312	−95.952	−31.392	179.76
M062X/aug-cc-pvdz	−143.92	−145.936	−138.368	−25.376	524.048
ωB97XD/6-311++G**	42.928	40.288	49.152	−29.728	4.64
ωB97XD/aug-cc-pvdz	−4.16	−6.224	1.344	−25.376	15.472
CCSD/6-311++G**	−77.296	−92.288	−50.16	−141.296	56.688
CCSD/aug-cc-pvdz	−81.2	−83.808	−74.352	−31.712	104.336

**Table 5:** Second order perturbation (E_(2)_) computation of the delocalization energies (kcal/mol) of 2-HPY, the transition state (T.S.) and 2-PY which were estimated by using CAM-B3LYP/aug-cc-pvdz level of theory.

Parameter	2-HPY	T.S.	2-PY
π_C1–C5_→π*_C2–N10_	20.42	0.974	1.45
π_C1–C5_→π*_C3–C4_	36.25	18.04	26.07
π_C2–N10_→π*_C1–C5_	38.71	0.504	0.50
π_C2–N10_→π*_C3–C4_	15.33	0.894	5.07
π_C3–C4_→π*_C1–C5_	21.85	9.10	16.61
π_C3–C4_→π*_C2–N10_	40.94	0.50	32.18
σ*_C1–N10_→σ*_C2–O11_	4.99	0.56	2.24
σ*_O11–H12_→σ*_C2–C3_	6.33	0.5	0.5
n_1N10_→σ*_C1–C5_	9.98	36.17	(55.80) #
n_1N10_→σ*_C2–C3_	12.49	0.45	0.5
n_1N10_→π*_C2–O11_	0.5	8.42	66.97
n_1N10_→σ*_C2–O11_	8.36	0.5	2.50
n_1O11_→σ*_C2–N10_	7.52	5.61	1.57
n_2O11_→π*_C2–N10_	43.08	0.50	35.24
n_2O11_→σ*_C2–C3_	0.5	4.51	20.57
n_2O11_→n*_H12_	-	70.11	-
n_2N10_→n*_H12_	-	100.36	-

# n_1N10_→π*_C1–C5_.

**Table 6:** Natural bond orbital (NBO) analyses of the total SCF, deletion and hyperconjugative energies (a.u.) for 2-HPY and 2-PY tautomers, which were estimated by applying CAM-B3LYP/aug-cc-pvdz//CCSD/aug-cc-pvdz level of theory.

Parameter	2-PY	2-HPY	ΔE ^a^
Total SCF Energy (Full)	−323.402843	−323.403258	−1.090
Energy of Deletion (L)	−322.535752	−322.566444	−80.582
Hyperconjugative Energy (NL)	−0.867091	−0.836814	+79.492

^a^ ΔE = E_2-HPY_ − E_2-PY_ kJ/mol.

**Table 7:** The Dipole moments (µ/Debye), HOMO and LUMO energies (eV) and their energy gaps (E.G. = ∆E/eV), and the total hyperpolarizabilities (β_tot_/a.u.) for 2-HPY and 2-PY which were estimated by utilizing B3LYP, CAM-B3LYP, M062X and ωB97XD functionals with 6-311++G** and aug-cc-pvdz basis sets. For comparison, the values for p-nitroaniline (p-NA) are given.

Level of Theory	Parameter	2-HPY	2-PY	p-NA ^a^
B3LYP/6-311++G**	Μ	1.464	4.506	7.17
	HOMO	−6.817	−6.349	
	LUMO	−1.135	−1.598	
	E.G.	5.682	4.751	4.290
	β_tot_	209.27	177.85	1327
B3LYP/aug-cc-pvdz	Μ	1.360	4.428	
	HOMO	−6.771	−6.312	
	LUMO	−1.133	−1.608	
	E.G.	5.638	4.704	
	β_tot_	203.55	195.01	
CAM-B3LYP/6-311++G**	Μ	1.523	4.556	7.23
	HOMO	−8.254	−7.756	-
	LUMO	0.172	−0.275	-
	E.G.	8.426	7.481	6.78
	β_tot_	197.44	149.25	1350
CAM-B3LYP/aug-cc-pvdz	Μ	1.415	4.427	
	HOMO	−8.198	−7.742	
	LUMO	0.173	−0.253	
	E.G.	8.371	7.489	
	β_tot_	192.10	162.10	
M062X/6-311++G**	Μ	1.480	4.456	
	HOMO	−8.155	−7.626	
	LUMO	−0.101	−0.541	
	E.G.	8.054	7.085	
	β_tot_	194.19	158.64	
M062X/aug-cc-pvdz	Μ	1.357	4.365	
	HOMO	−8.069	−7.645	
	LUMO	−0.111	−0.528	
	E.G.	7.958	7.117	
	β_tot_	204.14	181.59	
ωB97XD/6-311++G**	Μ	1.460	4.516	7.160
	HOMO	−8.773	−8.278	-
	LUMO	0.838	0.389	-
	E.G.	9.611	8.667	7.96
	β_tot_	200.14	150.31	1350
ωB97XD/aug-cc-pvdz	Μ	1.371	4.444	
	HOMO	−8.724	−8.241	
	LUMO	0.839	0.383	
	E.G.	9.563	8.624	
	β_tot_	198.65	167.58	
Expermintal ^b^	μ	1.39	4.26	
Experimental ^c^	β_П_(−2ω;ω;ω)	-	-	1072 ± 44

^a^ Taken from Reference [54]; ^b^ Taken from Reference [7]; ^c^ Taken from Reference [55].

## Supplementary Materials

Supplementary materials can be found at www.mdpi.com/1422-0067/17/10/1893/s1.
