# Supplementary material for: The Thermodynamic and Kinetic Properties of 2-Hydroxypyridine/2-Pyridone Tautomerization: A Theoretical and Computational Revisit
Source: Int J Mol Sci. 2016 Nov 14;17(11):1893. doi: 10.3390/ijms17111893 (PMC5133892; doi:10.3390/ijms17111893)
Supplement: Supplementary file 1 [file ijms-17-01893-s001.pdf]

Supplementary Materials: The Thermodynamic and Kinetic Properties of 2-Hydroxypyridine/2-Pyridone Tautomerization: A Theoretical and Computational Revisit

Safiyah A. Hejazi, Osman I. Osman, Abdulrahman O. Alyoubi, Saadullah G. Aziz and Rifaat H. Hilal

Table S1. Standard orientation of 2-hydroxypyridine (2-HPY) calculated by using CAM-B3LYP/aug-cc-pvdz level of theory.

Center Number	Atomic Number	Atomic Type	Coordinates (Angstroms)		
			X	*Y*	*Z*
1	6	0	−1.003643	−1.231244	0.000058
2	6	0	0.904003	0.030899	−0.000008
3	6	0	0.186126	1.237950	−0.000042
4	6	0	−1.201170	1.156316	−0.000007
5	6	0	−1.819476	−0.102518	0.000050
6	1	0	−1.437416	−2.233442	0.000105
7	1	0	0.716025	2.188796	−0.000091
8	1	0	−1.800651	2.067596	−0.000023
9	1	0	−2.903707	−0.203699	0.000081
10	7	0	0.338955	−1.173142	0.000033
11	8	0	2.260559	0.084668	−0.000070
12	1	0	2.573552	−0.833016	−0.000054

Table S2. Standard orientation of 2-pyridone (2-PY) calculated by using CAM-B3LYP/aug-cc-pvdz level of theory.

Center Number	Atomic Number	Atomic Type	Coordinates (Angstroms)		
			X	*Y*	*Z*
1	6	0	−1.059541	−1.191802	0.000000
2	6	0	1.064458	0.063913	−0.000001
3	6	0	0.249231	1.266037	0.000000
4	6	0	−1.118464	1.203656	0.000000
5	6	0	−1.807288	−0.048084	0.000000
6	1	0	−1.493091	−2.190520	0.000001
7	1	0	0.784351	2.213949	0.000000
8	1	0	−1.699035	2.128112	0.000001
9	1	0	−2.892873	−0.101857	0.000001
10	7	0	0.303930	−1.124328	0.000000
11	8	0	2.293296	0.004077	0.000000
12	1	0	0.856392	−1.974323	0.000000

Table S3. Standard orientation of pyridine (PY) calculated by using CAM-B3LYP/aug-cc-pvdz level of theory.

Center Number	Atomic Number	Atomic Type	Coordinates (Angstroms)		
			X	*Y*	*Z*
1	6	0	1.231090	−0.557746	−0.000046
2	6	0	−1.025456	−0.867754	0.000023
3	6	0	−1.282063	0.507596	0.000055
4	6	0	−0.190954	1.357671	0.000019
5	6	0	1.100074	0.821991	−0.000037
6	1	0	2.217440	−1.024352	−0.000092
7	1	0	−2.307722	0.869475	0.000103
8	1	0	−0.341407	2.437261	0.000035
9	1	0	1.980886	1.460596	−0.000069
10	7	0	0.187293	−1.395952	−0.000019
11	1	0	−1.856403	−1.541865	0.000069

Table S4. Some selected bond lengths (Å), bond angles and dihedral angles (degrees) of the optimized structures of 2-pyridone (2-PY) (top 11 lines) and 2-Hydroxypyridine (2-HPY) (bottom 11 lines) which have estimated by using different methods with 6-311++G** and aug-cc-pvdz basis sets. The crystal structure of 2-Pyridone and the microwave-normalized structure of 2-HPY are listed for comparison purposes.

Parameter		C2O11	N10C2	C2C3	N10C1	O11C2C3	N10C2C3	C2C3C4	O11C2C3C4	O11C2N10C1	N10C2C3C4
B3LYP	6-311++G**	1.223	1.411	1.451	1.364	127.0	113.0	121.6	−180.0	180.0	0.0
	Aug-cc-pvdz	1.230	1.411	1.452	1.365	126.9	113.2	121.5	−180.0	180.0	0.00
CAM-B3LYP	6-311++G**	1.220	1.399	1.449	1.362	126.6	113.3	121.5	−180.0	180.0	0.00
	Aug-cc-pvdz	1.225	1.399	1.450	1.363	126.6	113.5	121.4	−180.0	180.0	0.00
WB97XD	6-311++G**	1.220	1.401	1.451	1.361	126.7	113.2	121.5	−180.0	180.0	0.00
	Aug-cc-pvdz	1.220	1.397	1.448	1.357	126.7	113.2	121.5	−180.0	180.0	0.00
M062X	6-311++G**	1.220	1.402	1.453	1.362	126.7	113.1	121.5	−180.0	180.0	0.00
	Aug-cc-pvdz	1.220	1.398	1.449	1.358	126.7	113.2	121.4	−180.0	180.0	0.00
CCSD	6-311++G**	1.221	1.399	1.465	1.375	126.0	113.4	121.5	−180.0	180.0	0.00
	Aug-cc-pvdz	1.232	1.403	1.463	1.379	126.0	113.6	121.4	−180.0	180.0	0.00
Experimental [a]		1.252	1.379	1.437	1.362	124.8	115.0	121.1	−177.6	177.7	2.1
B3LYP	6-311++G**	1.354	1.326	1.401	1.341	118.5	124.0	117.4	−180.0	180.0	0.0
	Aug-cc-pvdz	1.353	1.323	1.397	1.338	118.6	123.9	117.5	−180.0	180.0	0.00
CAM-B3LYP	6-311++G**	1.348	1.319	1.395	1.336	118.5	124.0	117.4	−180.0	180.0	0.00
	Aug-cc-pvdz	1.351	1.323	1.399	1.339	118.5	124.1	117.4	−180.0	180.0	0.00
WB97XD	6-311++G**	1.346	1.322	1.398	1.337	118.5	124.1	117.4	−180.0	180.0	0.00
	Aug-cc-pvdz	1.350	1.326	1.401	1.340	118.4	124.2	117.3	−180.0	180.0	0.00
M062X	6-311++G**	1.348	1.321	1.399	1.338	118.3	124.2	117.4	−180.0	180.0	0.00
	Aug-cc-pvdz	1.351	1.324	1.402	1.340	118.2	124.3	117.3	−180.0	180.0	0.00
CCSD	6-311++G**	1.354	1.325	1.407	1.348	118.0	124.5	117.3	−180.0	180.0	0.00
	Aug-cc-pvdz	1.364	1.333	1.413	1.355	118.0	124.6	117.3	−180.0	180.0	0.00
Normalized [b]		1.336	1.320	1.41	1.34	118	124.1	117.5	-	-	-

[a] Taken from Reference [2]; [b] Taken from Reference [7].

Table S5. Standard orientation of the transition state (T.S.) calculated by using B3LYP/6-311++G** level of theory.

Center Number	Atomic Number	Atomic Type	Coordinates (Angstroms)		
			X	*Y*	*Z*
1	6	0	0.894017	−1.288327	0.000014
2	6	0	−0.959359	0.205596	−0.000009
3	6	0	−0.112054	1.335076	−0.000007
4	6	0	1.252633	1.090093	0.000005
5	6	0	1.778009	−0.219808	0.000016
6	1	0	1.214283	−2.325567	0.000022
7	1	0	−0.525865	2.336249	−0.000016
8	1	0	1.939885	1.932583	0.000007
9	1	0	2.847723	−0.393839	0.000026
10	8	0	−2.238063	0.037676	−0.000020
11	1	0	−1.714105	−1.235007	−0.000009
12	7	0	−0.425270	−1.050229	0.000002

Table S6. Imaginary frequencies (negative Signs) Harmonic frequencies (cm^{-1}), IR intensities (km/Mole), Reduced masses (amu), force constants (mDyne/Å), and normal coordinates calculated for the transition state (T.S.) by using B3LYP/6-311++G** level of theory.

**Imaginary Freq.**	−1889.5981			
**Reduced Masses**	1.0705			
**Force Constants**	2.2521			
**IR Intensities**	580.9988			
**Atom Number**	**Mass Number**	*X*	*Y*	*Z*
1	6	0.00	−0.01	0.00
2	6	−0.05	0.01	0.00
3	6	0.01	0.00	0.00
4	6	−0.01	0.00	0.00
5	6	−0.01	−0.01	0.00
6	1	0.00	−0.01	0.00
7	1	0.00	0.00	0.00
8	1	0.00	0.00	0.00
9	1	−0.01	−0.01	0.00
10	8	0.01	0.04	0.00
11	1	0.80	−0.59	0.00
12	7	−0.02	0.00	0.00

Table S7. Standard orientation of the transition state (T.S.) of the double proton shifts between a mixed dimer tautomerization of 2-HPY↔2-PY using B3LYP/6-311++G** level of theory.

Center Number	Atomic Number	Atomic Type	Coordinates (Angstroms)		
			X	*Y*	*Z*
1	6	0	2.016618	−1.540155	−0.000186
2	6	0	3.392801	−1.551071	0.000391
3	6	0	4.058686	−0.303647	0.000714
4	6	0	3.337370	0.866023	0.000434
5	6	0	1.910516	0.837407	−0.000207
6	1	0	1.443911	−2.460302	−0.000423
7	1	0	3.938541	−2.483769	0.000607
8	1	0	5.142238	−0.271514	0.001186
9	1	0	3.807521	1.838608	0.000673
10	7	0	1.293419	−0.389741	−0.000491
11	8	0	1.221139	1.938550	−0.000529
12	1	0	−0.000043	−0.416342	−0.000651
13	6	0	−4.058719	−0.303515	0.000683
14	6	0	−3.337346	0.866106	0.000410
15	6	0	−1.910460	0.837458	−0.000216
16	6	0	−2.016735	−1.540155	−0.000202
17	1	0	−5.142272	−0.271322	0.001144
18	1	0	−3.807455	1.838713	0.000643
19	1	0	0.000431	1.905944	−0.001177
20	8	0	−1.220960	1.938448	−0.000518
21	7	0	−1.293476	−0.389794	−0.000489
22	6	0	−3.392914	−1.550992	0.000364
23	1	0	−3.938712	−2.483656	0.000567
24	1	0	−1.444093	−2.460346	−0.000441

Table S8. Imaginary frequencies (negative Signs) Harmonic frequencies (cm^{-1}), IR intensities (km/Mole), Reduced masses (amu), force constants (mDyne/Å), and normal coordinates calculated for the transition state (T.S.) of the double proton shifts between a mixed dimer tautomerization of 2-HPY↔2-PY using B3LYP/6-311++G** level of theory.

**Imaginary Frequency**	−1310.7244			
**Reduced Masses**	1.0771			
**Force Constants**	1.0903			
**IR Intensity**	500.4067			
**Atom Number**	**Mass Number**	***X***	***Y***	***Z***
1	6	0.00	−0.02	0.00
2	6	−0.01	0.00	0.00
3	6	0.00	0.01	0.00
4	6	0.01	−0.01	0.00
5	6	−0.01	0.04	0.00
6	1	−0.01	0.00	0.00
7	1	−0.01	0.00	0.00
8	1	0.00	0.00	0.00
9	1	−0.01	0.00	0.00
10	7	−0.01	0.00	0.00
11	8	0.01	−0.02	0.00
12	1	0.83	0.00	0.00
13	6	0.00	−0.01	0.00
14	6	0.01	0.01	0.00
15	6	−0.01	−0.04	0.00
16	6	0.00	0.02	0.00
17	1	0.00	0.00	0.00
18	1	−0.01	0.00	0.00
19	1	−0.55	0.00	0.00
20	8	0.01	0.02	0.00
21	7	−0.01	0.00	0.00
22	6	−0.01	0.00	0.00
23	1	−0.01	0.00	0.00
24	1	−0.01	0.00	0.00

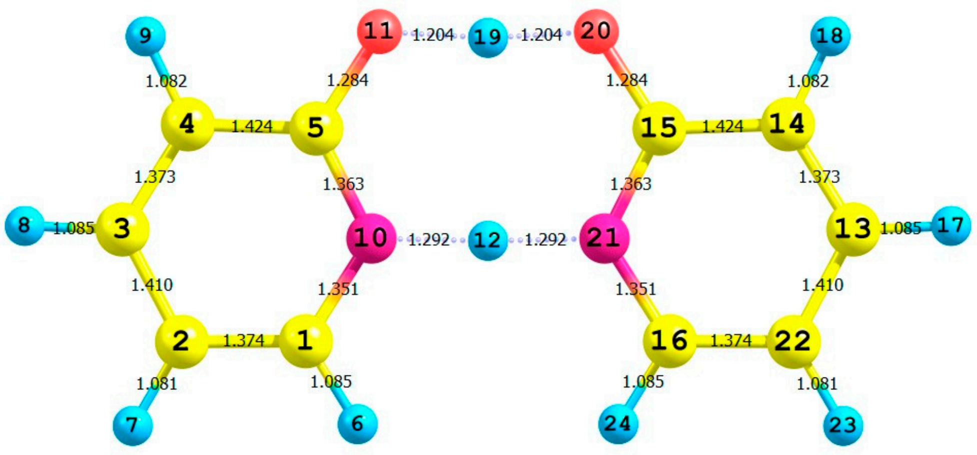

Figure S1. The structure of the Transition State for the double proton shifts between a mixed dimer tautomerization of 2-HPY↔2-PY using B3LYP/6-311++G** level of theory. The color scheme is pink: nitrogen; yellow: carbon; blue: hydrogen; and red: oxygen.
